# Action Experience, More than Observation, Influences Mu Rhythm Desynchronization

**DOI:** 10.1371/journal.pone.0092002

**Published:** 2014-03-24

**Authors:** Erin N. Cannon, Kathryn H. Yoo, Ross E. Vanderwert, Pier F. Ferrari, Amanda L. Woodward, Nathan A. Fox

**Affiliations:** 1 Department of Human Development and Quantitative Methodology, University of Maryland, College Park, College Park, Maryland, United States of America; 2 Laboratories of Cognitive Neuroscience, Division of Developmental Medicine, Children's Hospital Boston, Boston, Massachusetts, United States of America; 3 Dipartimento di Neuroscienze, Università di Parma, Parma, Italy; 4 Department of Psychology, University of Chicago, Chicago, Illinois, United States of America; UCLA, United States of America

## Abstract

Since the discovery of mirror neurons in premotor and parietal areas of the macaque monkey, the idea that action and perception may share the same neural code has been of central interest in social, developmental, and cognitive neurosciences. A fundamental question concerns how a putative human mirror neuron system may be tuned to the motor experiences of the individual. The current study tested the hypothesis that prior motor experience modulated the sensorimotor mu and beta rhythms. Specifically, we hypothesized that these sensorimotor rhythms would be more desynchronized after active motor experience compared to passive observation experience. To test our hypothesis, we collected EEG from adult participants during the observation of a relatively novel action: an experimenter used a claw-like tool to pick up a toy. Prior to EEG collection, we trained one group of adults to perform this action with the tool (performers). A second group comprised trained video coders, who only had experience observing the action (observers). Both the performers and the observers had no prior motor and visual experience with the action. A third group of novices was also tested. Performers exhibited the greatest mu rhythm desynchronization in the 8–13 Hz band, particularly in the right hemisphere compared to observers and novices. This study is the first to contrast active tool-use experience and observation experience in the mu rhythm and to show modulation with relatively shorter amounts of experience than prior mirror neuron expertise studies. These findings are discussed with respect to its broader implication as a neural signature for a mechanism of early social learning.

## Introduction

In the last decade the idea that action and perception may share the same neural code has been of central interest in social cognitive sciences, as well as in the neurosciences. This proposal has been stimulated by the discovery of mirror neurons in the ventral premotor cortex and posterior parietal lobule of the macaque monkey [1]–[3]. The fact that these visuomotor neurons discharge while the monkey performs an action and observes a similar action performed by another individual has led to the hypothesis that the visual perception of another's actions is mapped onto the internal motor representation of the observer. Thus, internal motor knowledge is exploited in order to translate the perception of another's actions into a motor format known by the individual, making possible the recognition (and understanding) of another's actions. These findings echo previous theoretical accounts [4]–[5] and enlighten more recent psychological theories of the links between action, perception, and mechanisms of social learning [6]–[9].

Mirror neurons, known for their characteristic execution-observation matching mechanism, also have the feature of selectivity. That is, they are not active to all movements one perceives but rather are attuned to acts that are goal-directed and within one's own motor repertoire. For example, monkeys are not typically tool users, and thus, mirror neurons studied in single cell recordings in the Rhesus macaque do not spontaneously respond to actions made with a tool. The initial recordings of mirror neurons demonstrated that the activation of mirror neurons was specific to grasping, reaching or placing actions executed solely by a hand but not in conjunction with a tool (e.g., pliers). It has been suggested that the observation of tool-use actions cannot be mapped onto the monkey's motor representation of these actions, because the monkey lacks the motor repertoire of tool-use, which results in their lack of motor representation of these actions [1], [10]. This initial work provided evidence for the selectivity of action execution matching, but also alluded to the idea that one's own motor experiences may play an important role in the function of a mirror system. Specifically, it has been proposed that neural mirroring provides a means to relate one's own motor representation to inform understanding the actions of others [11].

Another characteristic of mirror neurons that supports theoretical accounts of action perception links in behavior is their ability to adapt to new motor experiences. Ferrari and colleagues identified a group of mirror neurons that responded to observation of goal-directed tool use in monkeys after extensive motor exposure to the action with the tool [12]. More recently, neurons identified in the ventral premotor cortex were shown to become active during the performance of a tool action after first-hand training [13]. Consistent with some of the hand and mouth grasping mirror neurons that have been identified [2], these neurons also exhibit properties of generalizing to the goal of the action. Although the monkeys were trained on two different means to achieve the same goal of picking up an item with a tool, these mirror neurons discharged during both actions. Furthermore, after active training, these previously unresponsive cells became active during the observation of an experimenter performing these actions [14]. Taken together, these findings indicate the plasticity of the mirror system in monkeys during action and perception, and thus, suggest its potential as an important learning mechanism in development [15].

Similarly, an abundance of evidence from various imaging techniques supports the idea that the putative human mirror system is sensitive to motor expertise. Using fMRI, Calvo-Merino and colleagues [16]–[17] showed film clips of kinematically similar ballet and capoeira movements to professional ballet dancers, capoeira dancers, and novices. The dancers showed greater activation in areas associated with the mirror system (premotor and parietal areas) for the genre in which they were trained, whereas novices showed no differences in their neural response between genres. In a follow-up to this study, Calvo-Merino et al. [17] clarified that activation of parietal-premotor areas was not dependent on visual familiarity. Professional ballet dancers who viewed highly familiar yet gender specific ballet movements showed greater activation for movements performed by their own gender, i.e., to actions that were within their own motor repertoire. Indeed, these studies suggest the existence of relations between motor experience and the mirror system. However, they test expert dancers (and other specialists/athletes) with years of experience in their domain. Regardless, there is some evidence for plasticity based on shorter time scales of motor experience. Studies have examined how previous sensorimotor experience (e.g., novel drawing movements) during a small time window influences brain regions corresponding to the mirror system. Cross et al. [18] showed increased parietal/pre-motor activation in expert dancers who viewed movement sequences learned over a five-week period in comparison to unfamiliar movement sequences. Although the participants in their study were not as motorically naive as novices (i.e., they had general motor expertise within the domain tested), the findings suggest that the putative human mirror system can be modulated with only weeks of practicing a set of particular motor actions. Another study by Cross and colleagues [19] examined how the activity of the brain region involved in object manipulation (e.g., intra parietal sulcus) changed based on whether the participants were trained to only identify or learn to tie different knots. They found that this area was specifically activated during viewing of the various knots when the participants had sensorimotor experience with learning to tie the perceived knots. Together, these neuroimaging studies suggest that the putative human mirror system can be modulated in a short time window of practicing a set of particular motor actions.

On the other hand, a number of studies report findings in which motor experience does not result in greater activation of the mirror system. Instead, a decreased activation of the system was observed in line with the “neural efficiency” hypothesis, which posits that a more efficient cortical function is achieved with better performance in cognitive functions [20]. Del Percio and colleagues [21] for example found that elite karate athletes displayed reduced activation of the mirror system compared to non-athletes. A similar finding has also been demonstrated in a fMRI study by Vogt and colleagues [22]. Indeed, mixed results in the expertise mirror system literature make it difficult to form a coherent picture of the precise relation between motor experience and the plasticity of the mirror system in humans.

In the present study we tested the hypothesis that active motor experience with a tool within a relatively short period of time influences the activity of the mirror system in adults. We recorded EEG changes in sensorimotor alpha and beta bands. Characterized as the alpha band (8–13 Hz) at central electrodes, the *mu* rhythm desynchronizes both during observation and execution of goal-directed actions, reflecting mirror neuron function [23]–[24]. Though it is less clear where in the cortex the mu rhythm is generated, it has been proposed that the source is within the sensorimotor cortex [25]–[27]. Simultaneous fMRI and EEG recordings in adult subjects have shown that the mu-rhythm is associated with the activity of mirror neuron areas [25], [27], and therefore considered as a brain marker to investigate the role of experience and learning in modifying the activity of the mirror system [28].

Likewise, the *rolandic beta rhythm* (13–30 Hz at the centrally located sites) has also been shown to flutuate during the observation of others' goal-directed actions [29]–[32], and is related to one's prior motor experience [33]–[34]. For example, Järveläinen and colleagues [33] used median nerve stimulation and MEG on adult participants to show activation of the primary motor cortex via desynchronization of the beta rhythm during goal-directed acts made with a hand and a tool (chopsticks). Significantly less desynchronization was found in a third condition in which similar but non-goal-directed motions with the tool were observed. Moreover, the magnitude of the desynchronizatoin in beta related to the amount of prior experience the participants had with chopstick use. Furthermore, simultaneous EEG and fMRI recordings of the rolandic beta rhythm during action perception related to activity in primary motor cortex [27], suggesting it too, might be a neural marker for mirror neuron system activity.

Evidence spanning a range of action domains (e.g., karate, air rifle, music) has shown expertise effects on mu and/or beta rhythms during the execution or observation of actions within one's domain of expertise [35], [36]–[37]. Along the lines of the expert/novice fMRI work described above, EEG reactivity has also been explored in experts (e.g., expert dancers and non-dancers) [34], as well as in participants who received training on a short time scale. For example, Rüther and colleagues [38] visually familiarized participants to novel tool manipulations. They found that prior visual experience with tool manipulation modulated the activity of the mu rhythm where event-related desynchronization was greater for participants who received the training compared to those who did not. Quandt and colleagues [39] found that brief imitative experience with novel actions (novel hand drawings) led to greater desynchronization of alpha rhythm over the frontal regions. Another study by Quandt and colleagues [40] found that participants' prior somatosensory experience associated with an action modulated their alpha and beta rhythms over the frontal, central, and parietal regions during subsequent observation of the action performed by another person. It was also demonstrated that prior short-term sensorimotor experience with an object influenced alpha and beta rhythms and that this could affect how a gesture associated with a particular object is processed [41]. Together, these findings suggest that sensorimotor alpha and beta rhythms can be influenced by experience on a short time scale. However, an open question still remains concerning whether observational or active experience with a novel tool can differentially modulate the mu and beta rhythms.

In the current study, we tested the hypothesis that active experience, as opposed to observational experience, of performing an action modulates the mu rhythm during perception. This question differs from most prior EEG work because in the current context, experience was based on a much shorter time scale than experts who had years of training in their domain. Another difference between the current study and previous studies in expertise is that we examined mirror system activity (via mu and beta rhythm activity) during the learning of a general object-directed tool-use action. To note, previous studies with experts [18], [42] have investigated the mirror system activity during the learning of novel sequences of movements within their domain of expertise (not necessarily object-directed), whereas, we examined this using a more general action of tool-use. Moreover, to address the possibility that action familiarity is largely driving mu and beta reactivity in the central channels, we specifically tested the hypothesis that the physical experience of performing an action influences mu rhythm desynchronization during perception more than the experience of observing an action. To do this, we collected EEG while three separate groups of adults viewed trials of a person engaged in a tool-use action (picking up a toy with a mechanical claw). One group received prior training of using the tool to pick up each toy, a second group of adults received training on detailed observation of the tool actions being performed by someone else, and lastly a group of novices were tested, with no prior experience using the tool or viewing the particular toys. We analyzed the EEG signal in both alpha and beta bands across the scalp to determine whether any group effects were specific to central channels, or widely distributed across the scalp. If mu rhythm and beta rhythms are a reflection of mirror system activity, then the active experience group should exhibit greater desynchronization at central sites than the observation and novice groups. Furthermore, this pattern of desynchronization is not predicted to be robust at other sites across the scalp.

## Methods

### Ethics Statement

This study was approved by the Institutional Review Board at the University of Maryland, College Park. All participants were over the age of 18 and provided written informed consent before the study began.

### Participants

Thirty-three participants (8 males, 25 females) participated in the study. Of these, 12 (4 males, 8 females) were considered “novices,” (mean age = 20.14 years; SD = 1.46 years; undergraduates (*n* = 12)) entirely unfamiliar with the procedure and target action of the current experiment. These students were recruited from the Psychology department's online database. Eleven female participants were considered expert “performers,” (mean age = 20.36 years; SD = 0.92 years; undergraduates (*n* = 10), college degree (*n* = 1)) undergraduate staff in the lab, originally trained as experimenters in the procedure to grasp toys with a mechanical claw. All expert “performers” had performed the action a minimum of 150 times (*M* = 225.27; SD = 144.56) before their participation in this study. Ten participants (4 male, 6 female) were considered expert “observers,” (mean age = 23.00 years; SD = 5.21 years; undergraduates (*n* = 7), college degrees (*n* = 2), graduate degree (*n* = 1)) lab staff who had been trained in frame-by-frame video coding to identify the mechanical claw's first contact with the toy during the experimenter's grasp. These "observers" were highly familiar with the actions of the mechanical claw used by the presenter, as they had each coded a minimum of 200 trials (*M* = 401.50; SD = 213.36) prior to participation, but none had experience performing the action themselves. The number of trials for the “observers” and the “performers” groups was based on laboratory records and experimenter notes. Participant assignment in the “observers” and the “performers” groups was not entirely random as they consisted of laboratory staff. However, these participants were not recruited and placed in a group based on their coding and training performance. Although participants were not initially randomized to the different conditions prior to having only observational or active motor experience, all of the participants in the study were blind to the study's hypothesis. With these considerations, we felt that the current study was justifiable despite the lack of total random assignment of groups. An additional two novices participated but were excluded due to fewer than five artifact free segments per condition (*n* = 1), or were identified as statistical outliers whose event-related desynchronization/synchronization (ERD/ERS) values exceeded 3 standard deviations from the sample mean (*n* = 1). Participants in the “novices” group identified themselves as Caucasian (58.3%), African-American/Black (25%), Hispanic (8.3%), and Other (8.3%). Participants in the “performers” group identified themselves as Caucasian (81.8%), Hispanic (9.1%), and Other (9.1%). Participants in the “observers” group identified themselves as Caucasian (50%) and Asian/Pacific Islander (50%).

### Procedure

Three groups of participants were tested in this procedure: performers, observers, and novices. All participants were seated approximately 60 cm from the front of a small stage set up on a table (99 cm wide ×61 cm deep ×89 cm tall) covered with black cloth. A taupe curtain in front was raised and lowered for each trial. Areas immediately surrounding the stage were covered with black panel curtains to hide experimenters and equipment. A video camera at the back of the stage recorded the events of interest and participant behavior during trials.

**Figure 1:**
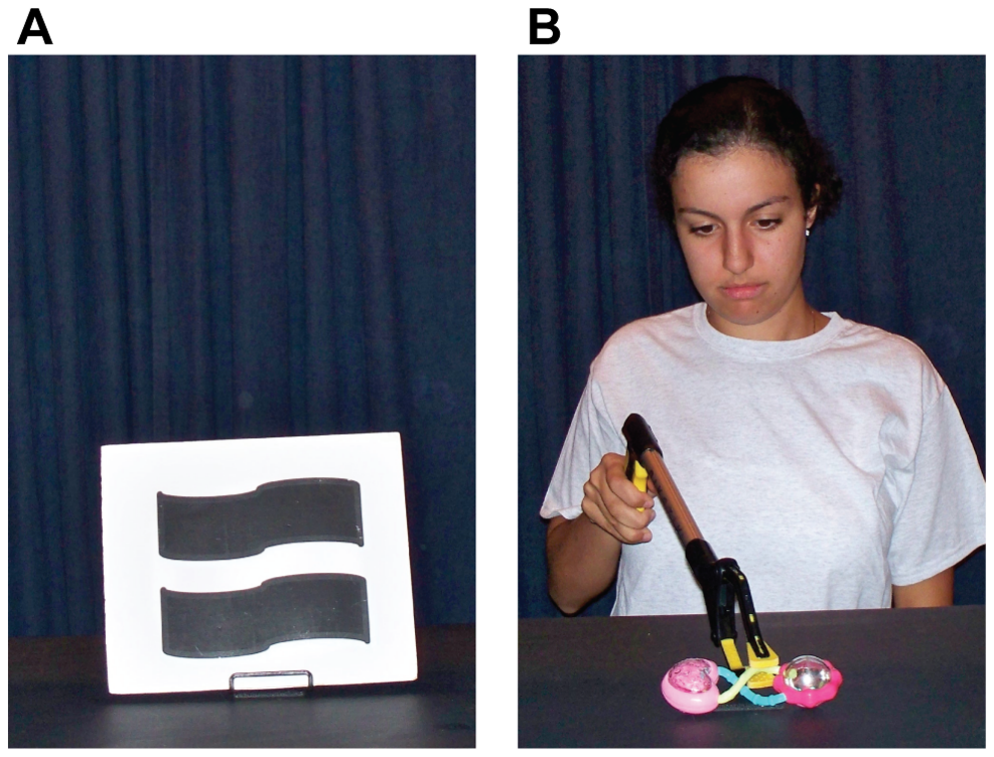
Example of the baseline stimulus (panel A) and the observation trial (panel B).

Each trial was preceded by a baseline period in which the curtain rose to reveal a black and white picture of a geometric shape (28×23 cm) for 3 s (Figure 1A), and then lowered. Then, the curtain rose again to reveal an experimenter sitting across from the participant and a toy at the center of the table. The presenter made brief eye contact with the participant, then shifted gaze to the toy, and then reached to pick it up using a mechanical claw-like tool (Figure 1B). The trial ended and curtain was lowered shortly after the toy was picked up and the action ceased. Participants were instructed to sit as still as possible and watch the pictures or events.

All participants completed 20 trials. Ten unique baseline pictures and ten small, colorful toys were presented. Each toy/baseline pairing was repeated in the second block of 10 trials. Pairings and orders were randomized across participants.

### Behavioral coding for EEG segmentation

Video that was synchronized to the EEG was recorded at a resolution of 640×480 and at a frame rate of 30 Hz. Two independent coders viewed each video offline (100% overlap), frame-by-frame and identified the frame in which the presenter made a touch to the toy with the tool that resulted in grasp completion. Inter-rater agreement within three frames (approximately 100 ms) was achieved on 92% of the trials. The EEG was then segmented around these event marks.

### EEG Acquisition and Analysis

EEG was recorded using a 64-channel HydroCel Geodesic Sensor Net and sampled at 500 Hz via EGI software (Net Station v4.5.1; Electrical Geodesics, Inc., Eugene, OR). Impedance values for all EEG channels were below 50 kΩ at the start of data acquisition. Electrodes placed above and below each eye were used to identify vertical eye movements. The EEG was referenced to the vertex during acquisition and re-referenced offline to an average reference. Channels 61–64, and channels 1, 5, 10, and 17 were contaminated by eye movements and removed prior to average referencing. The primary channels of interest with respect to mu rhythm desynchronization were the clusters that corresponded to central sites in the 10/20 system (C3: 15, 16, 20, 21, 22; and C4: 41, 49, 50, 51, 53). It has been suggested that C3 and C4 electrodes most likely represent sensorimotor activity during hand movements [43]. In the secondary analyses we present the data from Frontal (F3: 9, 11, 12, 13,14; and F4: 2, 3, 57, 59, 60), Parietal (P3: 26, 27, 28, 34; and P4: 40, 42, 45, 46), and Occipital (O1: 35; and O2: 39) regions.

Initial EEG processing was completed offline using Net Station (4.5.1) software tools. Data were filtered at .3–100 Hz for purposes of artifact detection and eye blink identification. Data were segmented +/−500 ms surrounding presenter's toy touch, consistent with the timing of mirror neurons' typical activation during a grasp act [2]. For baseline segments, 1000 ms of artifact free data were selected from the initial 3 s recording. If the entire 3 s was artifact free, the first second was selected. Net Station algorithms for eye blink detection and artifact (channels exceeding +/−150 μV) were applied. All segments containing eye blinks and those in which 10% or more of the channels exceeded threshold were removed from further analysis. The remaining number of segments were (a) baseline: *M* = 19.71, *SD* = .67, and (b) observation: *M* = 17.63, *SD* = 2.22.

Event-related desynchronization/synchronization (ERD/ERS) was computed using the methods similar to those described by Pfurtscheller [44]: the original raw EEG was band-pass filtered for the alpha rhythm (8 to 13 Hz) or the beta rhythm (15 to 25 Hz) and then squared to produce power values (μV^2^). For all clean segments identified in previous steps, average power was computed in 125 ms bins across all one-second segments. Because no time-related oscillations were expected during baseline, average power was computed across the 8 time bins for one aggregated baseline score. Next, the natural log of the ratio (event power divided by the baseline power) was calculated [45] for the event power at each 125 ms bin. The binned ratios were averaged across the segments into one aggregated ERD value for analysis. Values less than zero indicate desynchronization (ERD) and values greater than zero indicate synchronization (ERS).

### Data analysis

To examine whether the ERD/ERS values for “novices,” “observers,” and “performers” significantly differed on sex, we conducted a Group X Sex multivariate ANOVA. We then employed an omnibus mixed ANOVA for between-subjects measure Group (novices, observers, performers), and repeated measures: Band (mu, beta) and Hemisphere (left, right) on mean ERD/ERS at the central sites. Bonferroni correction was applied to all subsequent contrasts to correct for multiple comparisons. In addition, we implemented a Group X Time repeated measures ANOVA (group as between-subjects factor and time as within-subjects measure) to investigate group differences within the mu and beta bands as they change over time.

## Results

There were no significant Group X Sex interaction effects on all ERD/ERS values as revealed by a multivariate ANOVA (*for alpha band*: C3, *F*(1, 28) = 0.91, *p* = 0.35; C4, *F*(1, 28) = 0.06, *p* = 0.81; O1, *F*(1, 28) = 3.96, *p* = 0.06; O2, *F*(1, 28) = 0.00, *p* = 1.00; F3, *F*(1, 28) = 0.13, *p* = 0.72; F4, *F*(1, 28) = 0.18, *p* = 0.67; P3, *F*(1, 28) = 1.28, *p* = 0.27; P4, *F*(1, 28) = 0.16, *p* = 0.70. *For beta band*: C3, *F*(1, 28) = 2.34, *p* = 0.14; C4, *F*(1, 28) = 0.22, *p* = 0.64; O1, *F*(1, 28) = 1.47, *p* = 0.24; O2, *F*(1, 28) = 2.97, *p* = 0.10; F3, *F*(1, 28) = 0.00, *p* = 0.97; F4, *F*(1, 28) = 3.56, *p* = 0.07; P3, *F*(1, 28) = 0.18, *p* = 0.68; P4, *F*(1, 28) = 0.30, *p* = 0.59). There were also no significant differences in age between the three groups (*F*(2, 25) = 2.25, *p* = 0.13) The mixed ANOVA analysis revealed a Group X Hemisphere interaction, *F*(2, 30) = 8.77, *p* = .001, *η_p_^2^* = .37 and a marginal Band X Group interaction, *F*(2, 30) = 3.16, *p* = .057, *η_p_^2^* = .18. These interactions were followed up via planned comparisons of these variables in each individual band.

**Figure 2:**
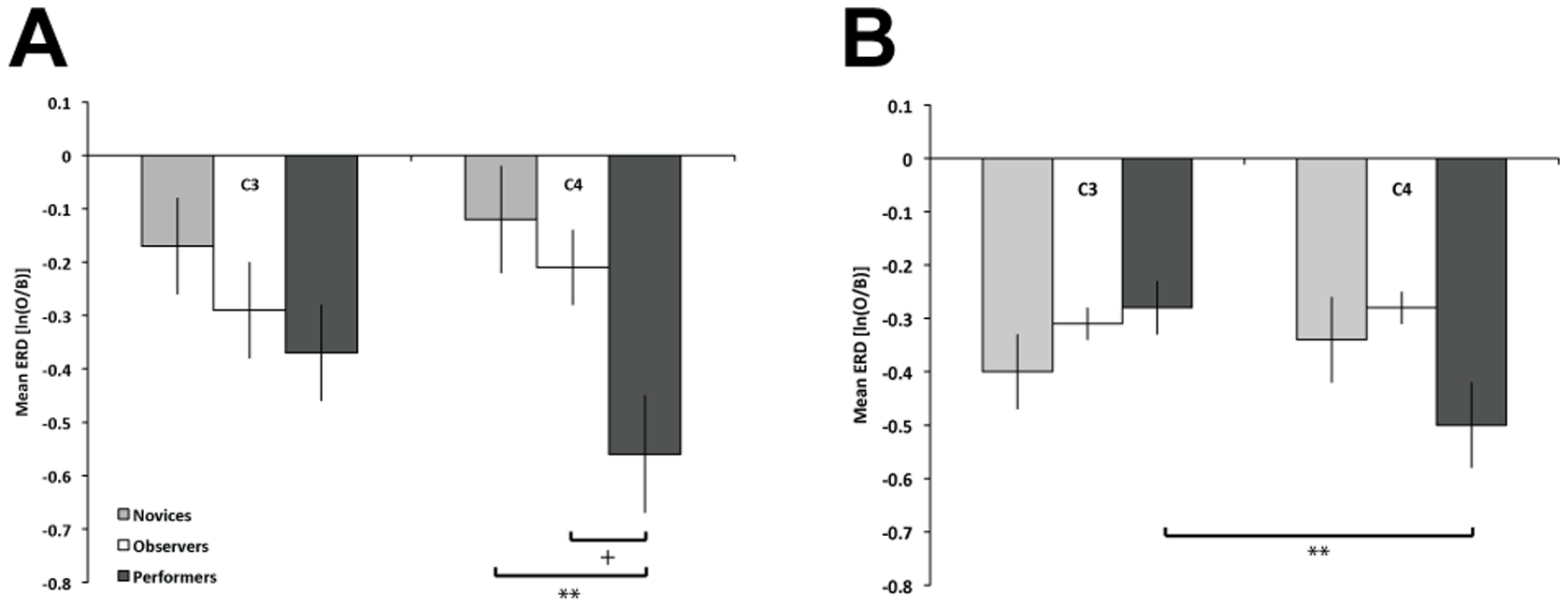
Mean ERD at C3 and C4 during tool use observation: *mu* band (panel A), and *beta* band (panel B).+ *p* = .05; ** *p*<.01.

Group means at each of the central electrode clusters are displayed in Figure 2A. The Group X Time repeated measures ANOVA revealed no significant effects of time and time differences between groups (time and group x time: *ps*>.55). There was a main effect of Group, *F*(2, 30) = 3.73, *p* = .036, *η_p_^2^* = .20 qualified by a Group X Hemisphere interaction, *F*(2, 30) = 5.13, *p* = .012, *η_p_^2^* = .26. Follow-up ANOVAs on each hemisphere indicated the group effect was most pronounced in the right hemisphere, C4: *F*(2, 30) = 6.15, *p* = .006, *η_p_^2^* = .26, rather than the left hemisphere C3, *F*(2, 30) = 1.41, *p* = .26. Post-hoc comparisons at C4 indicated the mean ERD of performers was more desynchronized than both novices (*p*<.006) and observers (*p* = .05).

**Figure 3:**
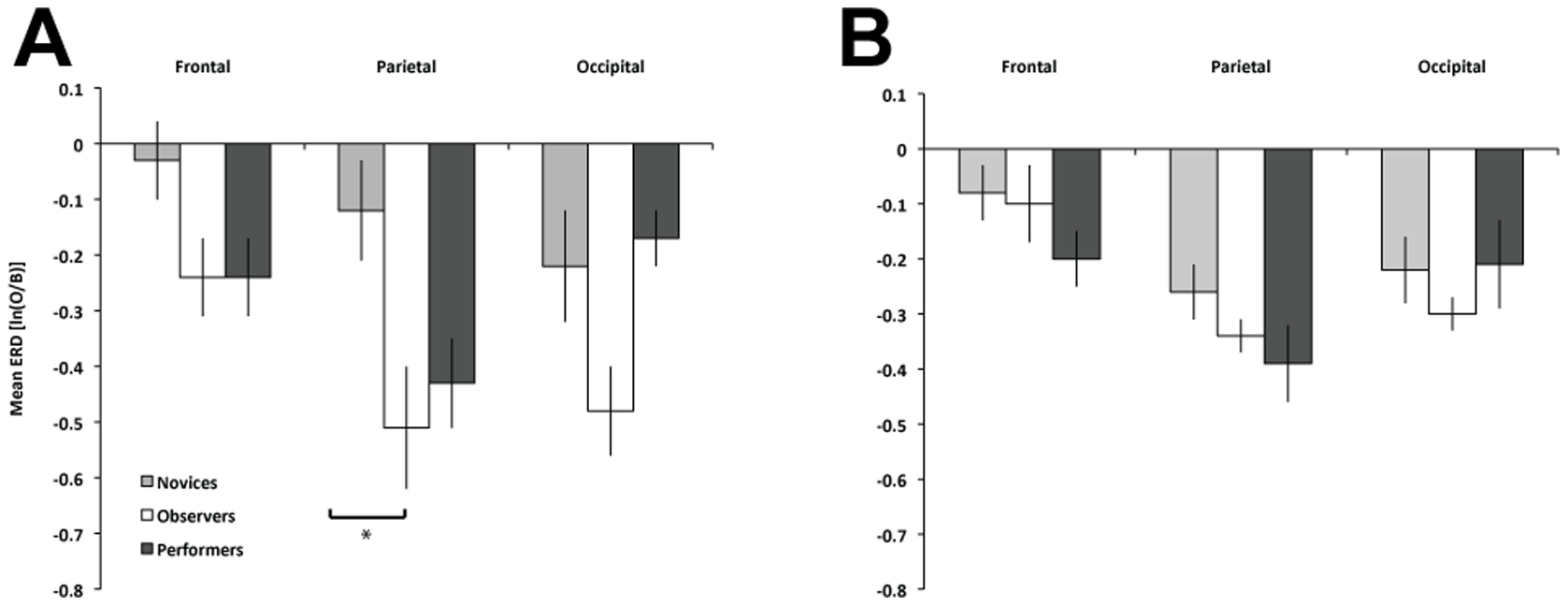
Mean ERD by group at frontal, parietal, and occipital sites during tool use observation: *alpha* band (panel A), and *beta* band (panel B). * *p*<.05, ** *p*<.01.

Group means in the beta band at each of the central electrode clusters are displayed in Figure 2B. The Group X Time repeated measures ANOVA revealed no significant effects of time and time differences between groups (time and group x time: *ps*>.05). There was a Group X Hemisphere interaction, *F*(2, 30) = 8.32, *p* = .001, *η_p_^2^* = .36. Although, follow-up comparisons of group in each hemisphere were not significant (*p*s>.10), the interaction stemmed from the performers, who showed overall greater ERD in the right hemisphere than the left (*p* = .005). Additional regions were analyzed for Group and Hemisphere effects on ERD/ERS in the alpha and beta bands. Group means for each additional region are displayed in Figure 3A (alpha) and 3B (beta). A Group X Hemisphere mixed ANOVA on Frontal ERD/ERS in the alpha band indicated a marginal effect of Group, *F*(2,30) = 3.18, *p* = .056, *η_p_^2^* = .18. Follow-up comparisons between groups were not significant (*p*s>.11). The same ANOVA on Parietal ERD/ERS in the alpha band revealed a main effect of Group, *F*(2,30) = 4.96, *p* = .014, *η_p_^2^* = .25, with ERD greatest for observers (*M* = −.51, *SE* = .10), who significantly differed from novices (*M* = −.12, *SE* = .09), *p*<.05, but not from performers (*M* = −.43, *SE* = .09), *p* = 1.0. The ANOVA on occipital region ERD in the alpha band also showed a significant effect of Group, *F*(2,30) = 3.95, *p* = .030, *η_p_^2^* = .21, though follow-ups were not significant (*p*s>.08).

A Group X Hemisphere ANOVA on Frontal ERD/ERS in the beta band revealed a significant effect of Hemisphere, *F*(1, 30) = 6.04, *p* = .020, *η_p_^2^* = .17. There was no significant Group X Hemisphere effect, (*p*>.05). Frontal beta ERD was greater in the right hemisphere (*M* = −.17, *SE* = .04) than the left (*M* = −.11, *SE* = .03). The ANOVA on parietal ERD/ERS in the beta band showed no effects or interactions (*p*s>.16). Lastly, the ANOVA on occipital beta ERD/ERS indicated a significant effect of Hemisphere, *F*(1, 30) = 6.80, *p* = .014, *η_p_^2^* = .19, such that occipital ERD was greater in the left hemisphere (*M* = −.29, *SE* = .04) than the right (*M* = −.21, *SE* = .06).

## Discussion

A fundamental question concerning the development of the human mirror system is whether active experience and/or passive viewing contributes to its plasticity. The findings reported here support the hypothesis that experience performing the tool-use action results in greater mu rhythm desynchronization more than observational experience (i.e., high visual familiarity) and no experience during the perception of this action. Mu rhythm reactivity reported here appears to be consistent with the characteristics of mirror system activity. Additionally, these findings further clarify that the mu rhythm can be modulated by active experience over a shorter time scale than has been previously used with EEG measures of experts and novices.

Our results showed greatest desynchronization of the mu rhythm in the group that received training in performing the tool use action in comparison to novices and experienced observers although the participants were not initially randomized to the different groups. This work is consistent with the mu rhythm findings showing that prior experience on a short time scale can modulate the mu rhythm [38]–[41], and with results contrasting expert and novice athletes, dancers, or musicians [21], [34], [37]. In studies with experts, however, it is difficult to control for group differences that may facilitate their expertise over novices. Furthermore, studies that investigate the relation between brief experience and mu rhythm activity have not examined how visual or active motor experience with a novel tool influences mu rhythm [38]–[41]. In our study, participants received training through active or observational experience with a tool use action (grasping an object with a mechanical claw). Using this method we were able to tease apart the contribution of differing levels of experience on mu and beta rhythm desynchronization.

One surprising finding was that our effect was strongest in the right hemisphere. Evidence from previous studies suggests that sensorimotor rhythms are lateralized during motor actions. Quandt and colleagues [41] report right hemisphere lateralization of sensorimotor rhythms in response to observed iconic gestures, which display characteristics of grasping and lifting an object. On the other hand, significant effects have been reported over the left hemisphere [40]. In addition, Del Percio and colleagues [46] report a relation between skilled karate performances and alpha ERD of the right hemisphere. Other studies examining the neural correlates of the mirror system have also reported right hemisphere effects (or lack of left-hemisphere lateralization) during perceptual tasks [47]–[48]. Orgs et al. [34] showed actions that involved movements of both arms and the torso to dancers and non-dancers. Although they did not statistically test for effects of hemisphere, but rather entered each individual electrode site into the analysis, their plots of the C3 (left) and C4 (right) sites for the mu rhythm band are suggestive of stronger desynchronization for the expert dancers in the right hemisphere (C4).

While studies examining the modulation of beta rhythm after short-term [40]–[41] and long-term [34] experiences report significant effects in this band, we did not find group differences in the rolandic beta band. We propose three possibilities for this discrepancy. First, lack of expert effects in the beta band may be a function of the shorter time scale of training that the participants received. Orgs et al. [34] tested dancers with approximately 15 or more years of experience, and [40]–[41] trained and tested participants in one experimental session. Our “expert” performers were trained and performed the action for approximately 9 months, with around 150 instances of performing the actions. Thus, the amount of experience of the participants in our study is somewhat moderate compared to these studies, potentially suggesting that beta rhythm activity may not remain stable over time during acquisition of sensorimotor skills. Our study narrows the window of experience needed to modulate the rolandic mu rhythm at central sites to a scale that can be measured on a scale of weeks to months of experience, but less than the more commonly used scale of years or single experimental session [16]–[17], [33]–[35], [38]–[41].

Another possibility is that reactivity of the beta band recorded over central sites may be more sensitive to the details of the kinematics and various properties of a presented stimulus. In the study by Orgs and colleagues [34], action sequences were complex in comparison to the action of reaching with the claw in the current work. They displayed dance movements, which were complicated sequences of movements that varied in movement velocities. Also, these movements were presented for a longer period of time (6–12 seconds) than our discrete reach to pick up a toy with a tool. In the studies by Quandt and colleagues [40]–[41], participants were exposed to objects that differed in their weight (i.e., heavy versus light). A study by Avanzini and colleagues [49] found diffferences in beta desynchronization between actions that were presented as one discrete act or were presented repetitively. Specifically, these differences in magnitude were related to the velocity profile of the actions viewed. Thus, if rolandic beta reflects an index for maintaining sequence and/or velocity profiles of actions, then group differences based on expertise may not arise in the context of the simple action we presented in this study. Issues of action velocity and sequence of more complicated actions in relation to motor expertise is therefore another avenue for future research that may further inform the potential function of the rolandic beta rhythm.

A third possibility for the lack of significant group differences in the beta band may be due to the differences in the neural cell assemblies that are involved in alpha and beta rhythms. Pfurtscheller and Lopes da Silva [50] have suggested that lower frequency rhythms comprise more neurons that are in synchrony compared to higher frequency rhythms. In another words, the amplitude of brain rhythms is proportional to the number of synchronous neural assemblies, thus, higher frequencies (e.g., beta) reflect more local brain activity (i.e., smaller number of synchronous neurons) compared to lower frequencies (e.g., alpha). Therefore, the lack of significant group differences in the beta component but evident in the alpha component suggests that more global neural assemblies may be involved in the learning of a general tool-use action.

As predicted, the effects of active motor training in the expert performer group were found at central sites and not at other locations across the scalp. Thus, our findings support the hypothesis that effects of motor expertise are selective to the central sites, and likely a reflection of motor activation during action perception. The only significant group difference at a non-central location was that the expert observer group showed stronger desynchronization than novices in the alpha band at parietal sites. The interpretation of this result is intriguing. The prolonged exposure of expert obervers to the task might have influenced attentional processes so that subjects were attending to visual features that were more relevant to their orginal coding task. Thus, it is possible that the advantages of observation training appears to have more influence on posterior sites, which may be more reflective of visual attention processes than cognitive or motor [42], [51]. It is also possible that central activity may be contributing differently to activity at parietal sites, which may explain the group differences we found between observers and novices [52]. However, it is not possible to conclusively determine how central mu activity is contributing to activity at other sites on the basis of our results.

The work presented here has direct implications for our understanding of the development of social cognitive processes and of the role of sensorimotor experience in modifying internal motor representations at the service of decoding others' behaviors. In particular, a growing body of evidence from behavioral work with human infants has found that changes in motor development of the infant can be tracked in relation to action perception, specifically their capacity to understand and or/anticipate others' actions [53]–[56]. Of particular interest are findings that the modification of infants' motor experience via active training appears to have direct effects on action perception, whereas observational training does not [57]–[58]. Although the current study does not directly test changes in an action-perception mechanism *per se*, it implicates a neural signature that is selectively modulated by action training and not via observation. Moreover, the data presented here suggest that the mu rhythm is reactive on a time scale that is viable to support changes in infant action perception, and thus, may play a role in support of social learning.

This early action-perception coupling system is being explored via EEG measures in infants. Fourteen- and sixteen-month-olds show greater desynchronization in a central region (Cz in this case), in the infant mu and beta bands when viewing other crawling or walking infants [59]. The magnitude of mu (7–9 Hz) and beta (17–19 Hz) desynchronization was related to the amount of active crawling experience of the infant. There is also evidence that daily training with a novel action may have effects on the mu rhythm band within the first year of life. Paulus and colleagues [60] showed effects as early as 7–8 months of age after receiving five minutes per day of active training with a rattle that produced a particular sound (action sound) and exposure to another object, which produced a sound (non-action sound). At test, infants showed greater desynchronization in the 6–8 Hz band after 1 week of training to the sound of the rattle that was associated with the action than to an equally familiar non-action or novel sound. Thus, there exists preliminary evidence that the mu rhythm is sensitive to active experiences within the first year of life, and may be modulated with as little as one week's experience. However, caution should be noted in this interpretation, as far less is known about the development of mu rhythm in relation to action experience and action perception in infants, particularly with respect to issues of frequency bands studied and scalp topography, see [61] for review.

There are a number of limitations to the current study. One concerns the method of group assignment and the use of laboratory staff in the observers and performers groups. Participant assignment to the observers and the performers groups was not entirely random (i.e., recruit from a random population, randomly assign to a group, and expose them to only observational or motor experience). However, to note, these participants were not aware of the study's hypothesis, and were not selectively assigned to a specific group based on their performance. Upon joining the laboratory, they were randomly appointed to a particular task of being a video coder or being a performer. Furthermore, the participants in the observers and the performers groups mainly consisted of undergraduates—the same pool of participants from which we would hypothetically recruit (as we did for the novices group). Another limitation concerns the difference in the amount of trials between the observers and the performers with observers having greater number of trials than the performers. It is possible that the amount of trial experience, whether observational or active motor, may be driving the reported effects. However, previous studies of expertise have reported *greater* desynchronization with *greater* experience [34], [62]. Based on these results, one would expect the observers in our study to show *greater* desynchronization compared to the performers since the observers had more trials than the performers. Nevertheless, the performers in our study showed greater desynchronization than the observers suggesting that active experience, more than observational experience influences mu rhythm desynchronization.

The study here contributes an important link between the developmental literature to-date and adult work that has largely indicated effects of motor expertise in the mu and/or rolandic beta rhythms. In particular, we showed that in adult subjects, the rolandic mu is sensitive to shorter amounts of experience than previously indicated by studies of motor expertise. It also opens the question of functional differences between mu and beta rhythm, such that rolandic beta may require a greater amount of experience or motor mastery than what was received here. Critically, in adults, we find that active experience, more than observational experience modulates the mu rhythm. Our finding provides support for a functional and a topographical neural marker that can be utilized in developmental studies of social learning and cognition. Thus, this study provides evidence consistent with what is known about the putative mirror neuron system and further provides a new approach to studying its development in humans: via active and observational training measures of EEG.
